# Rebound phenomenon and its risk factors after hemiepiphysiodesis using tension band plate in children with coronal angular deformity

**DOI:** 10.1186/s12891-022-05310-z

**Published:** 2022-04-08

**Authors:** Kug Jin Choi, Sanghoon Lee, Moon Seok Park, Ki Hyuk Sung

**Affiliations:** 1 Department of Orthopaedic Surgery, Seoul Now Hospital, Seongnam-si, Gyeonggi South Korea; 2 Department of Orthopaedic Surgery, Seoul National University College of Medicine, Seoul National University Bundang Hospital, Seongnam-si, Gyeonggi South Korea

**Keywords:** Coronal angular deformity, Hemiepiphysiodesis, Tension-band plate, Rebound phenomenon, Correction rate

## Abstract

**Background:**

This study was performed to evaluate the rebound phenomenon after the correction of coronal angular deformity by hemiepiphysiodesis using tension band plate in children and to identify its risk factors.

**Methods:**

We reviewed 50 children (mean age, 11.0 ± 2.5 years) with 94 physes who had undergone hemiepiphysiodesis using tension band plate due to coronal angular deformity of the lower limb. Patients’ demographic data including sex, age at initial surgery and plate removal, affected bone (distal femur or proximal tibia), affected side, and body mass index were collected. The mechanical lateral distal femoral angle (mLDFA) and the mechanical medial proximal tibial angle (mMPTA) were measured from the teleradiogram, Physes were divided into rebound and non-rebound group, and rebound group was defined as the physes which had  ≥  5° of mLDFA or mMPTA returning to its original deformity. Generalized estimating equation based multivariable analysis was used to identify the risk factors for the rebound phenomenon after the deformity correction.

**Results:**

A total of 41 physes classified into rebound group and 53 physes into non-rebound group. There were significant differences in the age at initial surgery (*p* = 0.004), the age at implant removal (*p* = 0.002), the amount of correction (*p* = 0.001), and the rate of correction (*p* < 0.001) between two groups. The rate of correction was significantly associated with the rebound phenomenon (*p* = 0.044). The risk of rebound phenomenon was 1.2-fold higher as the rate of correction increased by 1° per year. The cutoff values of the correction rate between the two groups were 6.9°/year (*p* < 0.001).

**Conclusions:**

This study showed that the rebound group had younger age and faster correction rate than those in the non-rebound group. In addition, the correction rate for deformity was a significant risk factor for the rebound phenomenon after hemiepiphysiodesis using the tension band plate. Close monitoring after implant removal is required for children who have a rapid correction rate over 7°/year.

## Introduction

Coronal angular deformity of the knee joint is a commonly diagnosed growth disorder in growing children. It may lead to cosmetic problems, gait disturbances, joint instability, and activity-related pain. Further, it may lead to early osteoarthritis due to abnormal joint overload [1].

Temporary hemiepiphysiodesis using staples, [2] percutaneous screws, [3] or tension band plate [4] is commonly used in growing children due to its effectiveness and fewer complications, such as delayed union and transient nerve palsy, as compared to corrective osteotomy [5]. Tension band plates function as a hinge on the convex side of the deformity and gradually correct limb alignment while preserving natural growth. Several studies reported favorable outcomes in terms of deformity correction and minimal hardware problem.[6–13].

After angular deformity correction, the implant is removed, and then, the inhibited side of the physis can continue to grow. However, the growth of the inhibited side of the physis can exceed that of the uninhibited side due to transient overstimulation, and this may lead to the recurrence of angular deformity after implant removal, which is a rebound phenomenon [14]. In case of severe rebound phenomenon, revision hemiepiphysiodesis or even corrective osteotomy may be required in skeletally mature patients [8]. Most studies advocate a slight overcorrection considering the rebound phenomenon [15, 16].

A few studies investigated the risk factors for the rebound phenomenon after hemiepiphysiodesis by tension band plate [17–19]. They showed that age, body mass index (BMI), degree of initial deformity, and correction rate were associated with the rebound phenomenon. However, previous studies have identified the risk factors by comparing the variables between the rebound and non-rebound groups, not by multivariable analysis, which could explain the effects of each risk factor on the dependent variables using s single statistical model [20]. Therefore, we performed this study was to evaluate the rebound phenomenon after the correction of coronal angular deformity by hemiepiphysiodesis using a tension band plate in children and to identify its risk factors using multivariable analysis.

## Materials and methods

 This retrospective study was approved by the institutional review board of Seoul National University Bundang Hospital, which waived the requirement to obtain informed consent. All methods were performed in accordance with the Declaration of Helsinki.

## Study population and data collection

**Fig. 1:**
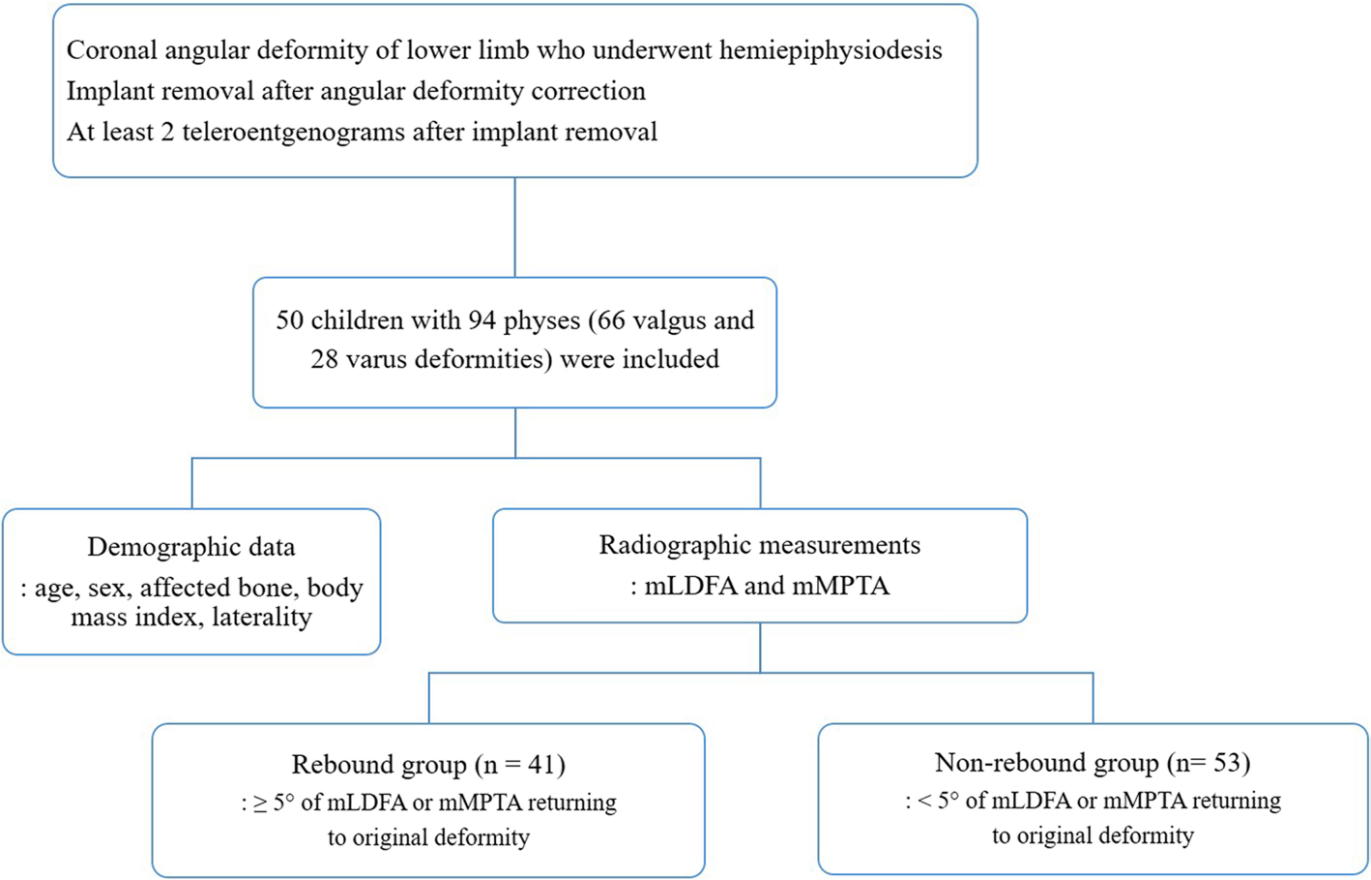
Flowchart for patients inclusion in this study. mLDFA, mechanical lateral distal femoral angle, mMPTA, mechanical medial proximal tibial angle

The inclusion criteria were: (1) consecutive patients with coronal angular deformity of the lower limb (genu valgum or genu varum) who underwent hemiepiphysiodesis using tension band plates between 2012 and 2019, (2) patients who underwent implant removal after angular deformity correction, and (3) patients who had at least 2 teleroentgenograms after implant removal. Patients who had inadequate preoperative or postoperative radiographs available for measurements were excluded (Fig. 1).

From the review of the medical records, we collected patients’ demographic data including sex, age at initial surgery and plate removal, affected bone (distal femur or proximal tibia), affected side, and BMI. For all patients, anteroposterior standing long-cassette radiographs of the lower extremity (teleradiogram) were taken using a UT 2000 X-ray machine (Philips Research, Eindhoven, The Netherlands) at a source-image distance of 200 cm (50 kVp, 5 mAs) with the patella facing forward. The teleradiograms were taken preoperatively and then postoperatively at 3 to 6 month intervals until angular correction. After implant removal, teleradiograms were also taken every 3 to 6 month until skeletal maturity. The radiographic images were retrieved using a picture archiving and communication system (PACS) (IMPAX, Agfa Healthcare, Mortsel, Belgium). The radiographic measurements were performed using PACS software.

## Surgical procedure

Hemiepiphysiodesis was performed at the site with the main angular deformity by two pediatric orthopedic surgeons (MSP and KHS). All patients were operated on using a single extra periosteal tension band plate (8-plate; BK Meditech Ltd., Hwasung, Korea) and two non-locking screws as described by Stevens [4]. Surgeons paid special attention not to damage the underlying periosteum. After surgery, a compression dressing was applied and daily activities were encouraged.

## Radiographic measurements

After literature review, the mechanical lateral distal femoral angle (mLDFA) and the mechanical medial proximal tibial angle (mMPTA) were selected for radiographic measurements. These radiographic indices reflected the joint orientation and the severity of deformity, and showed excellent interobserver and intraobserver reliabilities for measurements [21, 22].

**Fig. 2:**
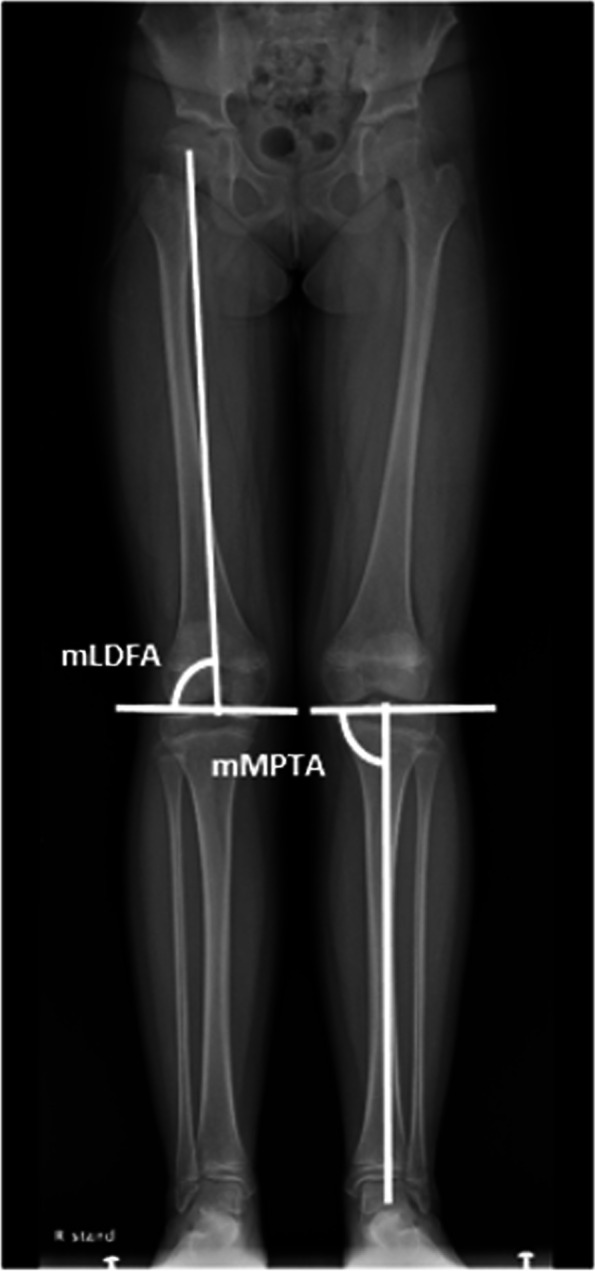
The mechanical lateral distal femoral angle (mLDFA) was defined as the angle formed by the line connecting the center of the femoral head and the center of the distal femoral epiphysis and the knee joint line of the femur. The mechanical medial proximal tibial angle (mMPTA) was defined as the angle formed by the line connecting the center of the proximal tibial epiphysis and the center of the talar dome and the knee joint line of the tibia

The mLDFA was defined as the angle formed by the line connecting the center of the femoral head and the center of the distal femoral epiphysis and the knee joint line of the femur. The mMPTA was defined as the angle formed by the line connecting the center of the proximal tibial epiphysis and the center of the talar dome and the knee joint line of the tibia (Fig. 2). We have defined the normal value of the mLDFA and the mMPTA as 88° and 87°, respectively [23]. The initial deformity angle was calculated as the absolute difference of the mLDFA/MPTA value from its normal value (88°/87°).

The inter-observer reliability was assessed before radiographic measurements were determined [24]. After estimating the sample size, 36 radiographs were randomly selected, and three orthopedic surgeons with 17, 6, and 5 years of experience, respectively, performed the measurement without knowledge of the patients’ clinical features and the measurements of the other two observers. After determining the inter-observer reliability, the main measurements of all radiographs were performed by a single author. For the physes which underwent revision hemiepiphysiodesis for recurrence of deformity after plate removal, radiographs before the revision surgery were measured. We divided the physes into rebound and non-rebound groups. The rebound group was defined as the physes which had ≥ 5° of mLDFA or mMPTA returning to its original deformity based on previous studies [18, 19].

## Statistical analyses

With an ICC target value of 0.8 and setting 0.2 as the width of the 95% confidence interval (CI), the Bonett approximation gave a minimum sample size of 36 for reliability testing [25]. The reliability of the measurement was attested by the intraclass correlation coefficients (ICC) and 95% CIs and were calculated in the setting of a two-way mixed-effects model, assuming a single measurement and absolute agreement [24]. An ICC value of 1 indicates perfect reliability and an ICC of > 0.8 indicates excellent reliability.

Descriptive statistics (mean, standard deviation, and proportion) was used to summarize patient demographics. Student’s t-test and chi-square test were used to compare the variables between non-rebound and rebound groups. To consider bilateral cases, a generalized estimating equation (GEE) logistic regression was employed. To identify the risk factors for the rebound phenomenon after the correction of coronal angular deformity, GEE-based multivariable analysis was used to calculate the adjusted odds ratios (ORs) and 95% CIs after univariable analysis. Variables with a *p*-value < 0.1 in the univariable analysis were included in the multivariable analysis. In the model, the patients’ age at surgery, sex, type of deformity, BMI, affected bone, initial deformity angle, rate of correction, and follow-up duration were treated as fixed effects, while the individual subjects and laterality were treated as random effects. The receiver operating characteristic (ROC) curve was used to define the cutoff values of the identified risk factors for the rebound phenomenon.

All statistical analyses were performed using the SAS statistical package, version 9.4 (SAS Institute, Cary, NC, USA), R (version 3.5.1) (R Foundation for Statistical Computing, Vienna, Austria. ISBN 3-900051-07-0, URL http://www.r-project.org), and MedCalc software for Windows (Version 18.11; MedCalc®, Ostend, Belgium). All statistical tests were two-tailed, and 95% CIs were considered significant when they did not include zero, and a *p*-value of < 0.05 was considered significant.

## Results

### Patient demographics

After the implementation of inclusion and exclusion criteria, 50 children with 94 physes were finally included; 66 were valgus, and 28 were varus deformities. The etiology of angular deformities was idiopathic, osteochondromatosis, posttraumatic, multiple epiphyseal dysplasia (MED), rickets, and focal fibrocartilaginous dysplasia (FFCD) in 79, 6, 5, 2, 1, and 1 physis, respectively. The mean rate of correction was 8.1 ± 4.7° per year, and the mean rebound angle was 4.2 ± 3.5°. A total of 41 physes classified into rebound group and 53 physes into non-rebound group. Revision hemiepiphysiodesis was performed in 8 physes (4 valgus and 4 varus deformities) for the recurred coronal angular deformity (Table 1). The inter-observer reliabilities for measuring mLDFA and mMPTA were excellent (ICC, 0.968 and 0.933, respectively).

**Table 1:** Summary of patients demographics

Variables	
Sex (male/ female)	47 / 47
Age at initial surgery (years)	11.0 ± 2.5
Body mass index (kg/m^2^)	20.5 ± 4.9
Type of deformity (valgus / varus)	66 / 28
Laterality ( right / left)	46 / 48
Site (distal femur / proximal tibia)	47 / 47
Duration of correction (year)	1.3 ± 0.6
Amount of correction (°)	9.1 ± 5.1
Rate of correction (°/ year)	8.1 ± 4.7
Follow-up after implant removal (years)	2.2 ± 1.2
Rebound angle (°)	4.2 ± 3.5
Rebound / non-rebound groups	41 / 53

## Comparison between rebound and non-rebound groups

There were significant differences in the age at initial surgery (*p* = 0.004), the age at implant removal (*p* = 0.002), the amount of correction (*p* < 0.001), and the rate of correction (*p* < 0.001) between rebound and non-rebound groups. Amount of correction and rate of correction were significantly higher in rebound group than those in non-rebound group. Age at initial surgery and implant removal was significantly younger in rebound group than that in non-rebound group. However, there were no significant differences in terms of sex, BMI, type of deformity, affected bone, initial deformity angle, and follow-up duration after implant removal (Table 2).

**Table 2:** Comparison of variables between rebound and non-rebound groups

	Non-rebound group (*N* = 53)	Rebound group (*N* = 41)	*P*-value
Sex (male / female)	28 / 25	19 / 22	0.677
Age at initial surgery (years)	11.7 ± 2.3	10.2 ± 2.5	0.004
Age at implant removal (years)	13.0 ± 2.4	11.3 ± 2.6	0.002
Body mass index (kg/m^2^)	21.4 ± 5.2	19.5 ± 4.3	0.058
Type of deformity (valgus / varus)	36 / 17	30 / 11	0.746
Site (distal femur / proximal tibia)	22 / 31	25/ 16	0.096
Laterality (right / left)	23 / 30	23 / 18	0.311
Initial deformity angle (°)	4.5 ± 4.2	3.8 ± 2.9	0.353
Amount of correction (°)	7.6 ± 5.6	11.0 ± 3.8	0.001
Duration of correction (°)	1.3 ± 0.7	1.2 ± 0.4	0.166
Rate of correction (°/ year)	6.5 ± 4.4	10.2 ± 4.3	< 0.001
Follow-up duration after implant removal (years)	2.0 ± 1.0	2.4 ± 1.3	0.145

## Risk factors for rebound phenomenon

GEE-based multivariable analysis showed that only the rate of correction was significantly associated with the rebound phenomenon after correction of the coronal angular deformity using the tension-band plate (*p* = 0.044). However, sex, age at surgery, BMI, type of deformity, the initial deformity angle, and affected bone were not associated with the rebound phenomenon. The risk of rebound phenomenon was 1.2-fold higher as the rate of correction increased by 1° per year (Table 3).

**Table 3:** Risk factors for rebound phenomenon after the correction of coronal angular deformity by hemiepiphysiodesis using tension band plate

Variables	Univariable analysis			Multivariable analysis		
	OR	95% CI	*P*-value	OR	95% CI	*P*-value
Sex (male / female)	1.4	0.5 to 4.0	0.474			
Age at initial surgery (year)	0.8	0.6 to 1.0	0.055	0.9	0.6 to 1.4	0.689
Body mass index (kg/m^2^)	0.9	0.8 to 1.0	0.071	0.9	0.8 to 1.0	0.192
Type of deformity (valgus / varus)	0.8	0.3 to 2.2	0.661			
Site (distal femur / proximal tibia)	0.4	0.2 to 1.3	0.134			
Laterality (right / left)	0.6	0.3 to 1.0	0.071	0.6	0.3 to 1.1	0.078
Initial deformity angle (°)	1.0	0.9 to 1.1	0.472			
Rate of correction (°/ year)	1.2	1.0 to 1.5	0.022	1.2	1.0 to 1.5	0.044
Follow-up duration (year)	1.3	0.9 to 1.9	0.226			
OR, odds ratio; CI, confidence interval						

**Fig. 3:**
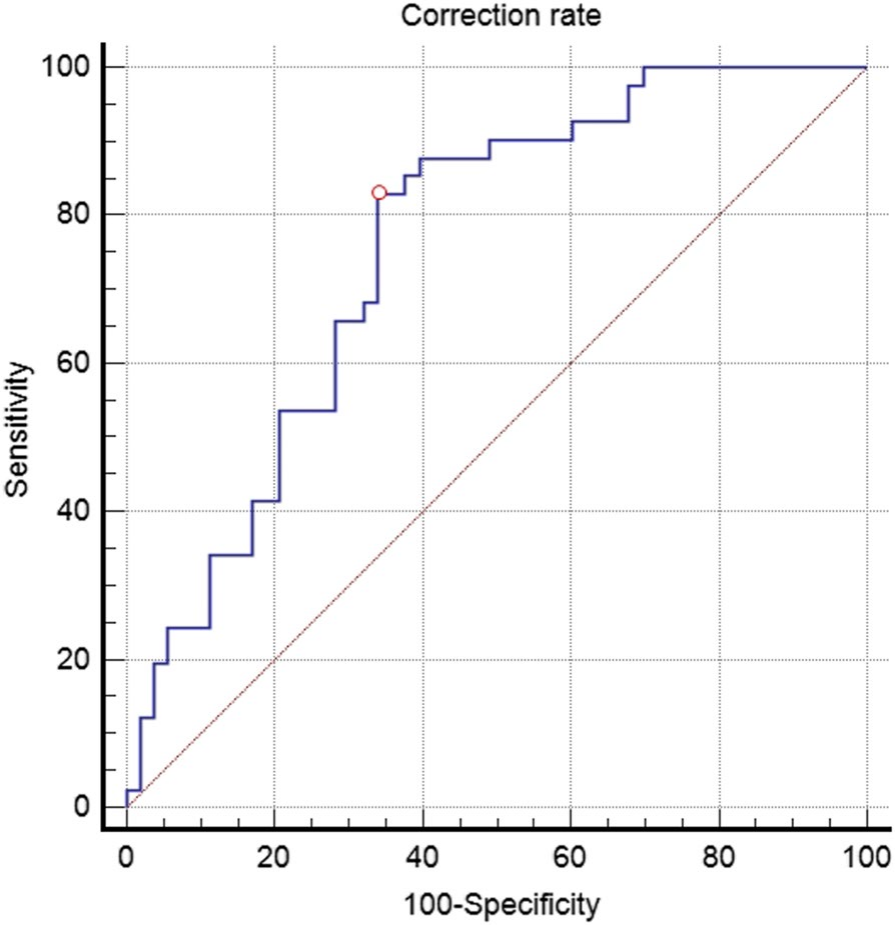
The receiver operating characteristic curves of the correction rate between the non-rebound and rebound groups are shown. This curve defines the cutoff value of correction rate as 6.9°/year

The cutoff values of the correction rate between the two groups were 6.9°/year (*p* < 0.001), and the ROC curve showed that the correction rate was a useful factor in determining the cutoff values for the rebound phenomenon (Table 4; Fig. 3).

**Table 4:** Cutoff value of the correction rate between the non-rebound and rebound groups

Parameter	Area of under the ROC curve	95% CI	*p* value	Cutoff value
Correction rate (° /year)	0.756	0.657 to 0.839	< 0.001	> 6.9

## Discussion

The present study evaluated the rebound phenomenon after the correction of coronal angular deformity by hemiepiphysiodesis using a tension band plate and identified its risk factors. Only the correction rate of deformity was a significant risk factors for the rebound phenomenon.

Stevens et al. reported a rebound of four cases out of 34 genu valgum cases with a mean duration of 14 months after plate removal; each patient electively underwent repeat guided growth with plates [4]. Kang et al. reported rebound phenomenon (rebound angle, ≥ 3°) after staple removal in 14 out of 37 physes cases, 11 of them (79%) experiencing a rapid rate of correction (correction angle per year, ≥ 8.5°/year) [26]. Leveille et al. reported 52% of patients with a rebound of the hip-knee-ankle angle of > 5° and 30% patients with a rebound of the hip-knee-ankle angle of > 10° after tension band plate removal in 67 physes. The results were relevant to severe initial deformity (≥ 20° from a neutral mechanical axis) and younger age at surgery and implant removal (< 10 years in girls and < 12 years in boys); both being found to increase the risk of rebound deformity [18]. Ramazanov et al. reported rebound phenomenon (rebound angle, ≥ 5°) in 56% of valgus deformities and in 24% of varus deformities. The rebound rate was higher in femoral valgus deformities, younger age at plate application and removal, higher correction rate of deformities, and intentional overcorrection [19]. Farr et al. showed children who are more than 1 year before skeletal maturity at plate removal or with increased BMI were high-risk for rebound phenomenon [17].

The rebound phenomenon appeared in 41 physes (44%) in our cohort. In this study, the correction rate was significant risk factor for the rebound phenomenon after plate removal. The finding that the higher correction rate resulted in an increased risk of the rebound phenomenon concurred with the results of previous studies [19, 26]. The correction rate of deformity is a direct indicator of physeal activity [26]. It indicates that if the rate of correction is higher, the residual activity of growth plate remains more, resulting in rebound phenomenon. In addition, the cutoff value of the correction rate for the rebound phenomenon was 6.9°/year. Therefore, surgeons should consider the high possibility of the rebound phenomenon after implant removal for patients with a correction rate of over 6.9°/year. This study showed that the initial deformity angle was not associated with the rebound phenomenon, despite previous studies reporting an increased risk [18]. The rebound group had a smaller initial deformity angle than the non-rebound group, with no significant difference. The amount and rate of correction were significantly higher in the rebound group than in the non-rebound group. We think that a significantly younger age at initial surgery and implant removal in the rebound group may result in an intentional large amount of correction considering the risk of rebound phenomenon.

Our study showed that age at surgery was not a significant risk factor for a rebound phenomenon (*p* = 0.689), which was different from the results of previous studies [18, 19]. However, the age at initial surgery and at implant removal was significantly younger in the rebound group than in the non-rebound group. In addition, age at initial surgery and at implant removal was negatively correlated with the rebound angle (correlation coefficient of − 0.404 and − 0.422, respectively). The younger the patient undergoes treatment, the higher the growth plate activity, the more rapid the correction rate, and the longer the time between plate removal and skeletal maturity, thereby increasing the risk of rebound phenomenon. Therefore, surgeons should carefully monitor the rebound phenomenon after implant removal if deformity correction is achieved at a younger age. In this study, there was no significant difference in sex between rebound and non-rebound groups and sex was not significant risk factor for the rebound phenomenon, as with the results of previous study [19].

Overcorrection has been recommended to prevent the rebound phenomenon. Park et al. recommended overcorrecting late-onset tibia vara by passing the mechanical axis 1 − 2 cm lateral to the knee center before staple removal [15]. Zuege et al. also recommended allowing 5° of overcorrection to allow rebound deformity [16]. However, not all children having the risk of rebound phenomenon actually undertake this process. Excessive overcorrection without rebound can lead to other types of coronal angular deformities and can be the cause of corrective osteotomy in a skeletally mature child. When a rapid modulation is identified, overcorrection within the clinically acceptable range and removal of the metaphyseal screw alone by leaving the plate and epiphyseal screw could be an option. When rebound takes place, simply reinserting the metaphyseal screw will start over the temporary hemiepiphysiodesis process, and this could be even less invasive than the first surgery.

There are some limitations to this study. First, the follow-up period after implant removal was not constant, which might have affected the results and follow-ups until skeletal maturity were not performed for all patients. However, there was no significant difference in the follow-up duration between the two groups and follow-up duration was not associated with the risk of rebound phenomenon. Further prospective studies with a scheduled long term follow-up until skeletal maturity are required. Second, we used a chronological age for statistical analyses due to the retrospective design of the study. Bone age is an indicator of the skeletal and biological maturity of an individual [27]. The use of bone age for statistical analysis will be able to provide consistent and reliable results. Further studies using bone age for risk factor analysis are required. Third, we did not consider the etiology as a variable affecting the correction rate. Despite including various etiologies, such as idiopathic, posttraumatic, MED, rickets, and FFCD, there were not enough patients for each etiology allowing for statistical analysis. Further studies with a sufficient sample size for each etiology are needed. Fourth, several studies showed that coronal knee alignment in adolescents was associated with participation in weight-bearing sports [28, 29]. Therefore, the degree of physical activity may affect the rebound phenomenon. Further study regarding the association between physical activity and rebound phenomenon could provide more guidance for patients.

## Conclusions

In conclusion, this study showed that the rebound group had younger age and faster correction rate than those in the non-rebound group. In addition, the correction rate for deformity was a significant risk factor for the rebound phenomenon after hemiepiphysiodesis using the tension band plate. Close monitoring after implant removal is required for children who have a rapid correction rate over 7°/year.

## Data Availability

The datasets used and/or analysed during the current study available from the corresponding author on reasonable request due to ethical concerns.

## Abbreviations

mLDFA: mechanical lateral distal femoral angle
mMPTA: mechanical medial proximal tibial angle
BMI: Body mass index
ICC: Intraclass correlation coefficients
GEE: Generalized estimating equation
